# Novel two-dimensional materials based bio-nanophotonics

**DOI:** 10.1515/nanoph-2022-0734

**Published:** 2022-12-08

**Authors:** Taojian Fan, Han Zhang

**Affiliations:** Department of the First Affiliated Hospital, Institute of Microscale Optoelectronics, and Otolaryngology, Shenzhen Second People’s Hospital, Health Science Center, Shenzhen University, Shenzhen 518060, China; College of Health Science and Environmental Engineering, Shenzhen Technology University, 518118, Shenzhen, China

In recent years, the novel diagnosis and treatment strategies based on optics aroused extensive interest of researchers and shown great clinical potential [1]. Due to its high sensitivity, optical technology has been used for single molecule level biodetection [2]. In the field of bioimaging, fluorescence imaging is one of the most common imaging methods, which can achieve high-resolution and non-destructive imaging [3]. In recent years, photoacoustic imaging with deep *in vivo* imaging ability and Raman imaging with high-throughput imaging potential also attracted extensive attention of researchers [4, 5]. In addition to biological detection and imaging, optical technology also possesses great potential in treatment. Due to the excellent time and space specificity of laser, phototherapy has the advantages of concentrated energy and slight side effects [6]. In fact, photodynamic therapy has achieved clinical transformation. While many ophthalmic treatment strategies are based on photothermal. Notably, the premise of most optical based diagnosis and treatment methods is efficient light response. Therefore, appropriate photosensitizers are crucial for the clinical transformation of novel optical diagnosis and treatment strategies.

Among numerous photosensitizers, two-dimensional (2D) materials have become a rising star due to their unique physical and chemical properties [7]. With ultra-thin planar structure, 2D materials exhibit a large specific surface area and thickness dependent optical response capability. In the field of detection, 2D materials can effectively capture the biomarkers and improve the signal intensity [2]. In the field of imaging, some 2D materials show strong photoacoustic signals, which are suitable for imaging [8]. In the field of therapy, a lot of 2D materials have high photo-thermal conversion efficiency and photodynamic quantum yield due to their excellent light response ability [9]. These developments show the great potential of biophotonics based on 2D materials in the biomedical field. This special issue on “Novel two-dimensional materials based bio-nanophotonics” outlines some of the latest developments in this topic through reviews and research articles.

Wang et al. [10] reviewed the potential of carbon nanomaterials in phototherapy, while Liu et al. [11] and Deng et al. [12] focused on the application of transition metal carbides and nitrides (MXenes) in biosensing, molecular Imaging and nanotechnology respectively. Francis et al. [13] discussed the prospects of 2D materials in personalized medicine, while Shivananju et al. [14] focused on the potential opportunities of nanophotonic in virus detection. In order to avoid the influence of tumor hypoxia microenvironment on photodynamic efficacy, Dang et al. [15] reported the oxygen self-supply engineering 2D materials for single-NIR laser-triggered synergistic photodynamic–photothermal therapy. Li et al. [16] proposed a strategy to promote the photodynamic performance of black phosphorus by generating oxygen through electrotherapy, while Wang et al. [17] designed an NIR-II light-activated I-type photodynamic strategy for the therapy of hypoxic tumors. Du et al. [18] showed a kind of multidrug strategy based on selenium nanoparticles for synergistic immunotherapy of osteosarcoma. Wang et al. [19] studied the self-powered broadband photodetector based on MoS_2_/Sb_2_Te_3_ heterojunctions for highly sensitive detection. Liu et al. [20] developed a fluorescein-derived carbon dots with chitin-targeting for ultrafast and superstable fluorescent imaging of fungi. Gong et al. [21] designed a pH-sensitive liposomal for fluorescence imaging guided intracerebral hemorrhage therapy. Dutta et al. [22] analyzed the magneto-optical behavior of the doped 2D CdSe nanoplatelets in the presence of paramagnetic copper ions. While Hu et al. [23] combined the nanoparticle-on-mirror family with the philosophy of remote spectroscopy to construct so-called “NPoM pairs” structures, which can be used for sensitive biodetection. Fu et al. [24] designed mesoporous carbon spheres with relative uniform pore channel arrangement and long pore channels for NIR light-activated drug release. An et al. [25] designed a manganese-functionalized MXene for MRI guided synergetic photothermal/chemodynamic therapy of cancer; while Zhang et al. [26] found that the Cu modified MXene exhibits the photothermal enhanced photodynamic properties under the NIR II irradiation.

In conclusion, this special issue provides introductions, reviews, and current research articles covering the 2D materials based bio-nanophotonics with a focus on photothermal, photodynamic, and fluorescent imaging. We hope that this collection of articles serves as inspiration for students and young as well as established researchers.
